# Government Food Pamphlets

**Published:** 1921-05-21

**Authors:** 


### Government Food Pamphlets.

It is to be hoped that Sir Alfred Mond meant more than he said in the House of Commons the other day in reply to Colonel Sir Arthur Holbrook, who asked him to state "when it is proposed to issue the educational food leaflet, the draft of which was submitted early in March, with a memorial on this subject from medical, scientific, health, and other associations," and whether he would deal with the matter as one of urgent importance, "having regard to the present general distress and the consequent necessity for disseminating knowledge of healthy, nourishing, and economical foods." The ministerial reply was as follows: "The matter is receiving attention, and I will see whether any useful purpose can be served by issuing any such pamphlet." This is the type of cautious official answer which of course conveys nothing, and is meant, one supposes, to convey nothing. But the question involved is so simple, and yet of so much importance, that there can be no adequate excuse for departmental delay. Several large public organisations, in addition to those whose representatives signed the original memorial, have urged the need, rendered the more necessary by the present distress, for disseminating the required information; and nearly a hundred members of the House of Lords and the House of Commons, representing all parties in Parliament, have issued a direct appeal to the Ministry to take action. We have seen no arguments put forward officially against distributing this useful information, the possession of which might in time improve the health and reduce the living costs of thousands of British families. There could be no more opportune moment than the present in which to act.

